# 

**Published:** 1852-05

**Authors:** 


### CONTENTS

OF THE

### MEDICAL EXAMINER.

NEW SERIES—NO. LXXXIX.—MAY, 1852.

### ORIGINAL COMMUNICATIONS.

Notes of a Case of latent Phthisis Pulmonalis terminating suddenly in Pneumothorax. By F. W. Sargent, M. D., one of the Physicians to the Girard College, - - - - 273

Note on Sulphate of Beeberine. By Henry S. Patterson, M. D., Professor of Materia Medica in Pennsylvania Medical College, 277

Case of Strangulated Crural Hernia successfully treated by Chloroform. Communicated by Robert Burns, M. D., of Frankford, 280

Remarks on the Climate and Epidemic Diseases of Rangoon, Burman Empire. By John Dawson, M. D. Communicated by J. Remington, M. D., of Philadelphia, - - - - - 282

Report of Cases, in continuation of the Remarks on Epithelial Tumors and Cancer of the Skin. By J. Da Costa, M. D., of Philadelphia, - - - - - - 287

Philadelphia County Medical Society. Extra Meeting, April 13th, 1852. Discussion on Anæsthesia, - - - - - 298

Experiments proving that life may be preserved in Warm-Blooded Animals after the destruction of a considerable part of the Spinal Marrow. By E. Brown-Séquard, M. D., of Paris, - 321

CLINICAL REPORTS.

Clinic of the Jefferson Medical College. Service of Professor Dunglison. Reported by J. Aitken Meigs, M. D., - - 322

BIBLIOGRAPHICAL NOTICES.

A Treatise on the Diseases of the Chest; being a Course of Lectures delivered at the New York Hospital. By John A. Swett, M. D., Physician to the New York Hospital; Member of the New York Pathological Society, - - - - - 331

EDITORIAL.

Pennsylvania Medical College, - - - - - 339

MEDICAL NEWS.

Death of Professor Grant, - - - - - - 340

Naval Surgeons, - - - - - - - 340

Relative Mortality of the States, - - - - - 340

